# Exploring Underlying Features in Hidden Layers of Neural Network

**DOI:** 10.3390/s25185755

**Published:** 2025-09-16

**Authors:** Sreenivas Sremath Tirumala, Jacqui Whalley

**Affiliations:** 1 RMIT University, Ho Chi Minh City 700000, Vietnam; 2 Lincoln University, Lincoln 7647, New Zealand; jacqui.whalley@lincoln.ac.nz

**Keywords:** artificial neural networks, knowledge discovery, feature mapping

## Abstract

The black box nature of artificial neural networks limits the understanding of internal mechanisms and processes that happen inside hidden layers. The introduction of deep neural networks and efficient layer-wise training methods has enabled researchers to study how features are learnt through different layers of neural networks. However, there has been limited research on mapping input features to neural network weights in order to understand how features are represented in the layers. This research proposes a novel component model to establish the relationship between input features and neural network weights. This will aid in optimizing transfer learning models by only extracting relevant weights instead of all the weights in the layers. The proposed model is evaluated using standard IRIS and a set of modified IRIS datasets. Classification experiments are conducted, and the results are evaluated to verify the quality of the dataset. A visualization of input features and components through the proposed model is presented using t-SNE to indicate the impact of changes in the input features. From the results, it is concluded that the proposed component model provides core knowledge in the form of weights representing the input features that are learnt through training. The proposed work will aid in designing component-based transfer learning, which would improve the speed. Also, the components could be used as pretrained testing models for similar work with large datasets.

## 1. Introduction

The functionality of artificial neural networks (ANNs), in terms of how features are managed by weights in the layers, has been the subject of investigation since the early 1990s. With advances in hardware, the study of individual ANN layers became possible, and these studies provided information on how weights are organized based on input data [1]. There have been several attempts to explore ANN layers to understand how the features are represented in the ANN weights. The introduction of layer-wise training enabled ANNs to expand with sufficient layers (depth) to be known as deep neural networks or DNNs. This exploration enabled researchers to devise various approaches for the transfer of knowledge and the transfer of learning.

A key aspect of ANNs lies in their ability to learn. In the case of ANNs, their learning capability is related to the depth, or number of layers, of the neural network, and so DNNs provide more efficiency and accuracy than traditional ANNs with fewer layers. The learning capability of DNNs can only be evaluated through experiments, and conclusions are made based on the outcomes of those experiments.

Understanding the functionality behind deep learning has enabled researchers to come up with several approaches for knowledge transfer and transfer learning. Despite there being several published works on transfer learning, it was common for earlier researchers to use the terms knowledge transfer and transfer learning interchangeably. The key goal of knowledge transfer is to move knowledge to enable a warm start for a new classifier. In the case of transfer learning, the learning capability, which includes several parameters, is transferred so that ‘fine-tuning’ may not be necessary [2,3].

When performing knowledge transfer, the primary goal is to extract knowledge [4,5]. The process of extracting knowledge from ANNs involves understanding, identifying, and precisely defining the knowledge inside neural network layers. However, what constitutes knowledge in ANN layers remains a point of debate. Knowledge transfer from a trained neural network to a new ANN often involves copying weights from one layer of the original ANN to another layer in the new ANN or simply copying weights from a trained ANN to a new ANN, followed by a fine-tuning process, such as retraining with a subset of new input data [6,7].

This practice suggests an idealized model of knowledge in which every weight in a neural network layer constitutes or is a part of knowledge. However, “dropout”, a technique designed to prevent overfitting through removing neurons, has called this idealized view of neural network knowledge into question [8]. Further evidence that knowledge is not as simple as the combination of every weight in a neural network layer has been provided through work that used a “weights of weights” approach, in which only significant weights in a layer were transferred, rather than all the weights. The “weights of weights” approach was shown to provide a more accurate transferable model than the traditional approach of copying all layers [9].

This work led to the following questions:

- How is knowledge represented in neural network weights?
- Does the concept of least and most significant weights exist?

While there is no debate on the practical application and efficiency of knowledge transfer learning approaches, there are several questions around the theoretical understanding of the importance and contribution of individual weights. For instance, when weights or layers are transferred, assuming there are insignificant weights being transferred (as suggested by [8,9]), is there an effective way to identify and remove insignificant weights?

The research presented in this paper provides an experimental validation process designed to understand the impact of changes in the input features on the weights of an artificial neural network. This will help to isolate and extract a set of specific ‘significant weights’ as components that could be used in the transfer of knowledge.

A novel component-based model for correlating input features with neural network weights is presented and evaluated. The model is used to extract components denoted by a set of neural network weights to correlate the input feature vectors with neural network weights. Traditional transfer of knowledge relies on copying all the layers (all the weights) into a new untrained neural network. The novelty of this research is proposing a model to extract transferable knowledge components, which could improve efficiency compared to traditional approaches.

This paper is organized as follows: Section 1 introduces the research topic and outlines its significance. Section 2 provides background on neural networks and the principles of knowledge discovery. In Section 3, the proposed component-based model is detailed. Section 4 presents the experimental results along with a discussion of the findings. Finally, Section 5 concludes the paper and outlines directions for future research.

## 2. Related Work

### Neural Networks and Knowledge Discovery

One of the most prominent areas of research since the discovery of the success of deep learning has been that of knowledge discovery [10,11]. It seems evident from the literature that knowledge resides in the form of neural network weights, some being significant and others being insignificant. In early work on knowledge discovery, it was assumed that all the weights in the layers of the neural network constituted knowledge [1,12,13]. In essence, neural networks attain knowledge through training, which is provided by learning algorithms. When weights in the hidden layers are ‘sufficiently trained’, then the ANN becomes efficient. The success of deep learning is attributed to the way that learning is achieved through several hidden layers; thus, it might be reasonable to assume that all hidden layers are equally important. However, the weights in the hidden layers near the input layer of deep neural networks are not optimized as they are too input-specific. In contrast, the weights near the hidden layer of the classifier are fully optimized, better representing the characteristics that facilitate the classification process [14]. From this analogy, it can be postulated that the center region of the DNN represents the transformation, thus providing a bridge from hidden layers with input-specific feature weights and the output-specific hidden layer to the classifier.

A neural network is said to be trained if the weights in the hidden layers constitute a formation that represents knowledge through which a particular problem can be solved. The knowledge that is attained through training is transferred to an untrained neural network, which enables the latter to solve similar problems with minimum training since it already possesses the ‘required knowledge’ [14]. This study defines knowledge as follows: ‘knowledge in a neural network is the expertise attained by training’ [14]. This expertise is the understanding of the unique characteristics of features that are used to determine classes or categories of data. This knowledge is used to efficiently perform classification and prediction.

The relationship between input features and hidden representations in the weights (knowledge) will enable us to understand, to some extent, how knowledge is attained through training. It is to be noted that training is a form of fine-tuning hidden layers to achieve (identify) optimal weight values (and parameters in some cases, but overall weights are the key).

The knowledge that neural networks acquire through training is based on how efficiently the features are learned by neural networks. The representation of knowledge in the neural network weights is in the form of underlying representations [15,16]. The representations and their patterns are based on features present in the input. Neural networks ‘learn’ to identify features through underlying representations present in the hidden layer weights. This is reflected in the functionality of the transfer of weights from a trained neural network to an untrained neural network.

Therefore, the extraction of knowledge is the extraction of a set of weights (if not all weights) from (several) hidden layers. This is a regular practice in the transfer of knowledge. However, there are concepts like dropout that remove insignificant weights [8]. In addition, methods of merging multiple layers into single layers to constitute a new network have shown some success [9]. In a nutshell, the existing literature on the transfer of knowledge is based on extracting and transferring weights from a trained network to another network.

From the literature, it is evident that existing knowledge extraction or knowledge transfer approaches use a ‘bulk’ transfer of weights, regardless of whether they are significant or insignificant. In a neural network’s hidden layers, each weight constitutes some operational influence when the weights are fine-tuned. This operational influence impacts, significantly if not to some extent, the performance of the network. Also, the greater the number of weights, the longer it would take to operate (train and test) the network.

It is noteworthy to observe that concepts like dropout have significantly improved the performance of the network. This brings us to addressing insignificant weights and understanding the importance of extracting only significant weights that constitute knowledge and transferring them to an untrained network.

The set of significant weights that possess knowledge is named a ‘knowledge component.’ A knowledge component consists of significant weights (knowledge) that are attributed to the effectiveness (efficiency and accuracy) of the neural network.

Moving away from the traditional approaches of knowledge transfer and transfer learning, this research aims to explore the possibility of extracting only significant weights as a set of knowledge components that can be transferred to a new neural network to provide a warm start.

## 3. Component-Based Model

The extraction of neural network weights as transferable knowledge components is the key aim of this research. To perform this task, it is necessary to develop a procedure to identify significant weights that constitute knowledge. To start with, it is evident through previous studies that some layers of neural networks are more significant than other layers [1,14]. The identification of significant weights has been proposed and tested by the author in earlier publications. The challenge here is to extract a set of weights that constitute knowledge from the layers and transfer these weights to a new untrained neural network. The relationship between input features and neural network weights can be established by studying the weight components in the layers.

The presence of significant knowledge in the middle layers was discussed and presented in [1,14]. To identify and extract significant knowledge components (a set of weights that constitute knowledge is a knowledge component), it is necessary to understand the distribution of knowledge among various layers of neural networks.

### 3.1. Proposed Model

According to the literature and earlier experiential works, it is evident that

- Layers near the input consist of features that are raw and low-level, as discrete pieces that reflect a form of input.
- The layers near the output are high-level representations that are very similar to the class labels responsible for the classification.
- The transformation of features (the learning mechanism) occurs between the input and output layers.

### 3.2. Components and Factor Analysis

A component is considered a set of features as defined in the popular statistical approach of Factor Analysis (FA) and Principle Component Analysis (PCA). The concept of extracting components from weights can be aligned with the extraction of components from features, as proposed in FA. It is important to note that PCA is a special case of FA. Statistical approaches like FA have the ability to determine the number of components based on variance, since this research is based on extracting neural network weight values.

PCA is able to extract components based on data in the dataset (considering various parameters) and is widely accepted since it eliminates any bias since the process is automated based on a strong statistical model. Also, each component is based on the projection of input features (dimensionality reduction), which is an ideal case for extracting significant weights as a combination of weights (set or component). FA is defined as a domain-specific PCA based on the assumption (or strong rule) of data from a specific category. PCA has the ability to associate features with components for various domains by identifying and mapping the underlying relationships [17,18]. The unique capability of PCA (FA) is its ability to identify and project hidden patterns that cannot be determined by clustering. This makes PCA the best possible approach to extracting components from the weights. There are several implementations of PCA and FA that are predominantly based on features and feature extraction [19,20,21].

### 3.3. Extracting Components

To extract components, it is necessary to answer the following questions.

- Should all the layers be used for component extraction?
- How can the number of components or any exit criteria be determined to make sure sufficient knowledge is extracted?

Several theories have been developed for the learning of neural networks [1,9]. A set of experiments were conducted in [1,9]. They show that there are some layers that possess weights that contribute significantly to the learning of neural networks. Considering layer-wise knowledge determination experiments, as provided in [1,9], it is evident that middle layers possess significant knowledge that is attained by neural networks through training.

The middle layers consist of significant weights that constitute knowledge responsible for the overall efficiency of neural networks [9]. Hence, it is appropriate to extract knowledge components from the middle layers.

Every input consists of a set of features to learn. Each input feature is represented by one or more attributes in the form of a linear combination. Consider a set of *n* input features defined as *F*, represented by

(1) $$F=f_{1},f_{2},\ldots ,f_{n}$$

Each input feature is represented in the neural network weights (nodes of the layers).

Consider a neural network with *L* layers, i.e., l=1 for the first layer and l=1…L (total number of layers). An input feature is represented as $f_{i}$. The knowledge constituted by weights can be extracted as multiple components. For instance, a layer *l* may have *n* components formed by different combinations of weights. $C_{li}$ represents the $i_{th}$ component in layer *l* with correlated weights *w* and error ϵ.

The features thus represented in weights are the projection of attribute-based input features, as presented in a study on the Blind Signal Separator Model [22]. The combination of input values and training variables (in this case, neural network variables like error, training algorithm variable, etc.) constitutes the projection of input features in the neural network layers.

A component *C* to be extracted from the weight tensors *W* can be defined as

(2) $$C=F^{\prime }S+\varepsilon$$

where

$F^{\prime }$ represents the projection of features *F* as a vector component;
ϵ is the stochastic error;
*S* represents the underlying functional values responsible for changes in the transformation of input features into components.

A neuron is a component comprising a set of weights (projection) from the neural network that represent one or more input features. In other words, $F^{\prime }$ is the projection of *F* to the features that exist as underlying features in the neural network weights *W*. It should be noted that the weights *W* are optimized through training, providing a clear projection of *F*.

*S* is introduced to consider the fact that all neural network weights consist of underlying features, i.e., not all weights are significant. The underlying features (significance) exist in a group of weights including some individual weights that are not significant, which is practically proven by concepts like dropout [8,23]. Therefore, it can be stated that the value of *S* will be high for a neural network with a high classification error. In other words, if the neural network is properly optimized for classification, the value of *S* will be minimal or, in ideal situations, zero.

Now, the key task is to represent $F^{\prime }$ in terms of *W*, the neural network weights that constitute *S*.

To start with, a reliable model for extracting components from a set of values (attributes or features) is PCA. Since *W* is represented as values (node weights), it will be appropriate to extract feature components from *W*.

Thus, with PCA, $F^{\prime }$ can be obtained as a covariance matrix of *W*.

Therefore,

(3) $$F^{\prime }=W\mu$$

where *W* is the weight vector and μ is the variance.

By substituting the value of $F^{\prime }$ into Equation (2), we obtain

(4) $$C=W_{\mu }S+\varepsilon$$

where *C* gives the generalised value of a component in a neural network.

To obtain a specific component *o* in layer *l*, we can use

(5) $$C_{l,o}=W_{o}\mu_{l}S_{l}+\varepsilon_{o}$$

Thus, Equation (5) presents a specific component in a particular layer.

The component presented in Equation (5) demonstrates the limitations of PCA, which is efficient for non-overlapping features. For complex and overlapping features, an alternative approach would be Factor Analysis (FA). In fact, PCA is a special case of FA with non-overlapping features.

Using the FA model from statistics, Equation (5) for the first component (o=1) can be written as

(6) $$C_{l,1}=F_{1}^{\prime }S_{l,1}+F_{2}^{\prime }S_{l,2}+\cdots +\varepsilon_{l}$$

Now, the generalization of Equation (6) for a set of features comprising a component *a* in a layer *l* can be written as

(7) $$C_{l,a}=\sum_{p}F_{n}^{\prime }S_{l}+\varepsilon_{l}$$

#### Determining Components

The extraction of components using PCA or FA has been mathematically proven to be successful and efficiently implemented for various types of datasets. However, determining the number of components that are extracted or, in some cases, need to be extracted can be achieved through similar approaches using PCA and FA. PCA transforms the correlated variables into components based on total variance. On the other hand, FA uses the commonalities that are summarized into a consolidated value to determine and extract factors.

The experiments for this research are performed on overlapping features. Therefore, the components can be determined as follows:

(8) $$C_{j,a}=\sum_{p}F_{n}^{\prime }S_{j}+\varepsilon_{j}$$

where

$F_{n}^{\prime }$ is the factor based on variance.

From Equation (8), all possible components can be extracted from that particular layer.

## 4. Experiments Results and Discussion

The aim of these experiments is to demonstrate the proposed component model through knowledge transfer. Transfer learning approaches often involve transferring all layers or parameters from a trained neural network to an untrained one, as seen in [24,25,26,27]. To evaluate the successful implementation of the proposed model, the experiments are divided into two parts. First, components are extracted, and then the efficiency of the model is assessed through transfer learning experiments.

A key aspect of the proposed model is that the features are represented through the weights of a neural network, and these sets of weights constitute transferable knowledge components. Data visualization is a widely accepted method for analyzing data, as highlighted in [28,29].

### 4.1. Evaluating the Relationship

This section presents an evaluation on how neural network classification is affected by changes in input features. This will serve as an initial attempt to isolate components for further experiments. The IRIS dataset is considered one of the best datasets for feature- and taxonomy-based experiments using machine learning algorithms [30].

**Figure 1:**
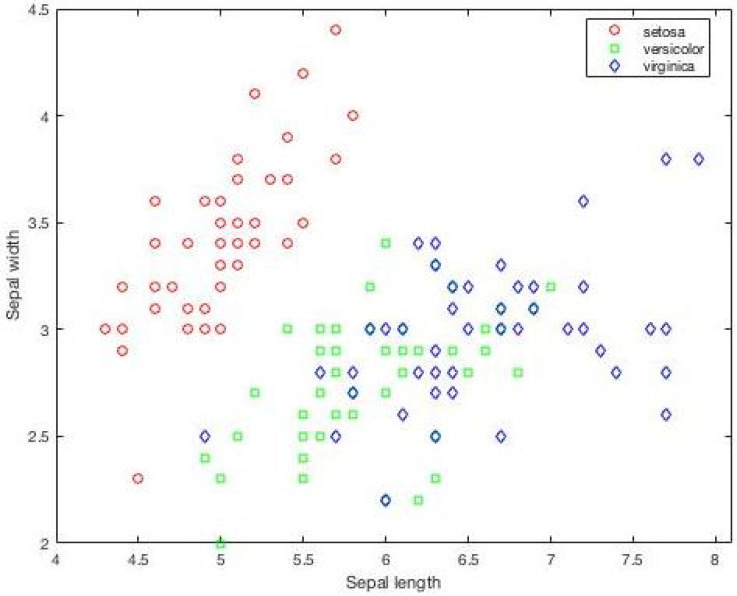
Visual representation of IRIS classes showing the distribution of individual values based on classes.

**Figure 2:**
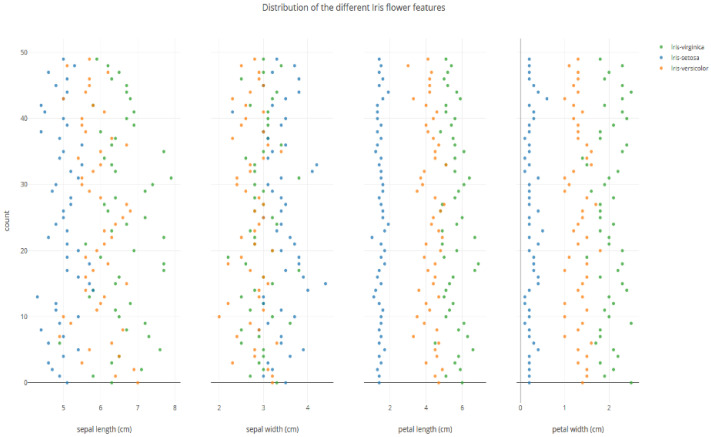
Visual representation of individual attributes and their values represented in colors based on the classes they belong to.

The IRIS dataset consists of 150 samples with four attributes and three classes. The visualization of the IRIS dataset based on clusters is presented in Figure 1. Also, it is important to plot the individual data points in the IRIS dataset for each attribute to understand the overlapping of values. The initial positions of the values of various attributes are presented in Figure 2. The sepal length and sepal width attributes are closely associated for all classes. The petal length and petal width for one class show a clear separation, as presented in Figure 2. The classification accuracy of IRIS is 99%; one sample contains values that are nearly identical and associated with two classes.

**Figure 3:**
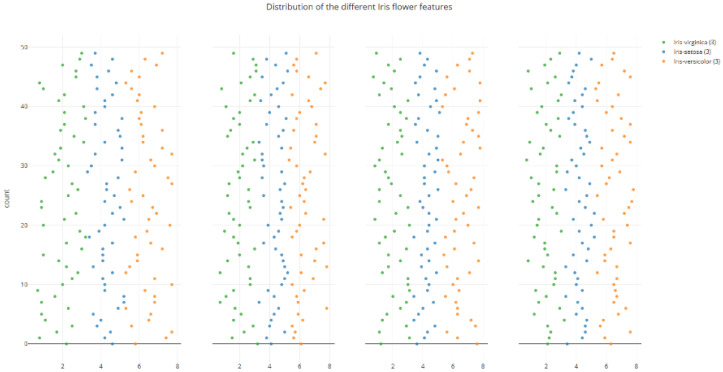
Modified IRIS: Visual representation of individual attributes and their values represented in colors based on the classes they belong to.

A new dataset, M-IRIS, is created by modifying some of the values to control the overlapping of features. Also, the M-IRIS dataset is tailored to achieve an ideal accuracy of 100%. Figure 3 represents the visualization of the M-IRIS dataset. In Figure 3, it can be seen that there is overlap for all four attribute values.

Comparing the neural network weights using IRIS and M-IRIS would indicate how weights are modified by slightly changing the attribute values in the input for the same dataset.

Three variants are produced for the M-IRIS, named MIRIS1, MIRIS2, and MIRIS3. The original IRIS dataset is modified to achieve an accuracy of 100%, and the new dataset is called MIRIS1. MIRIS2 and MIRIS3 are obtained by adding an additional attribute to MIRIS1. For MIRIS2, synthetic random values are generated to ensure that there is no correlation with the original four attributes. In the case of MIRIS3, the added attribute will be filled with values such that it produces a correlation with the other four attributes.

The components were extracted using PCA and FA modeling using MATLAB.

A variety of neural networks with 3, 5, 9, and 13 layers and a fixed number (30) of hidden nodes were used for the classification experiments. The experiments were carried out using MATLAB and Weka, with an average run of 30 each. The stochastic gradient descent (SGD) algorithm was used for training, with a learning rate of 0.32 and a momentum of 0.48. The experiments were carried out for 500 epochs with 70% and 30% for training and testing and three-fold cross-validation. The experimental results presented are averaged out.

The results from the classification experiments are presented in Table 1, Table 2, Table 3 and Table 4 for the IRIS and modified IRIS datasets, respectively. It can be noted that a three-layered neural network was able to produce the highest accuracy for the original and modified IRIS datasets, including the addition of a new attribute. However, in a special case, MI1 produced the highest accuracy for a five-layered neural network as it required a bit more depth to learn overlapping features.

The *t*-test and the RMS value are used to determine the statistical significance of the experimental results in terms of understanding their reliability. The *t*-test values are used to determine the uncertainty between the individual results obtained in different executions. The *t*-test values also give us the consistency of the results from the individual runs since the experiment results, accuracy in this case, are averaged for multiple runs.

The root mean square (RMS) error, sometimes called as the RMSE, emphasizes the average magnitude of the prediction error, i.e., the magnitude of error occurring between the expected results and the obtained results. Typically, a higher value signifies that the experiment has reached its maximum capability. A lower RMS error directly implies better accuracy. The truthfulness of the experiment results can be evaluated based on this pattern (the known relationship between RMS error and accuracy).

For MIRIS2 and MIRIS3, i.e., additional attributes with and without overlapping features, it can be noted that there is a huge 14% difference for the three-layer neural network.

**Figure 4:**
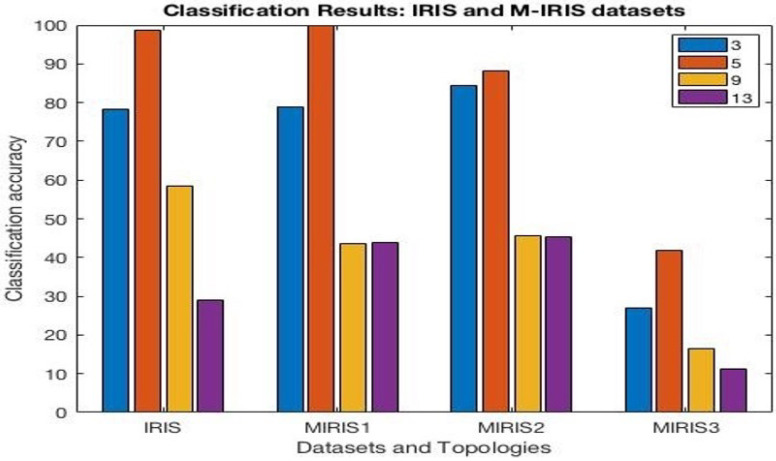
Classification results: Experimental results for IRIS and MIRIS datasets for different numbers of layers (3, 5, 9, 13).

From the experiment results shown in Figure 4, it is important to note that increasing the number of layers may not always improve accuracy. The depth, going deeper into layers, will increase the segregation of features into more deeply valued levels, which makes it difficult to learn, as that level of discreteness may not be required. It can be noted that modified IRIS MI1 was able to achieve 99% accuracy, as expected. The *t*-test (near zero) results and RMS error (directly proportional to accuracy) signify the reliability of the experimental results.

As shown in Figure 4, the experimental results illustrate that the changes in the input data were clearly reflected in the learning mechanism, with changing accuracy based on the type of change. It is noteworthy to observe that there is a clear difference between IR2 and IR3, indicating the impact of adding attributes that are correlated and non-correlated.

### 4.2. Exploring Components

The theoretical concept of the components existing in the neural network weights could be established through visualising neural network weights in the middle layers of a trained neural network [31]. T-distributed stochastic neighbour embedding can be used for visualising neural network weights to see how the features are represented in the weights.

**Figure 5:**
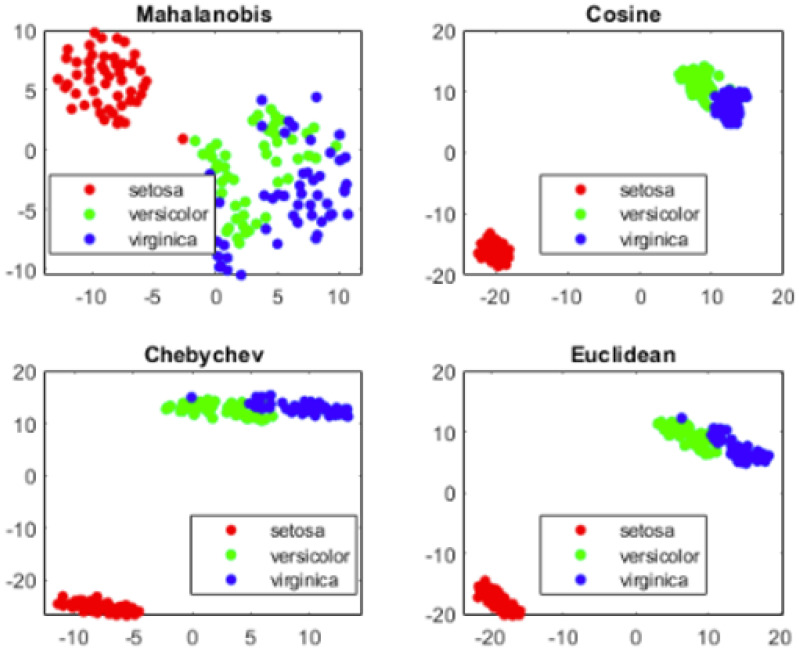
Visualization: Depiction of IRIS dataset with three classes representing Euclidean distance to show the isolation of setosa.

The visualization presented in Figure 5 shows the IRIS dataset with four different distance measurement approaches used to depict classes. Distance measures enable us to understand how classes are clustered based on the values. This will provide confirmation of the possibility of extracting components from neural network weights.

**Figure 6:**
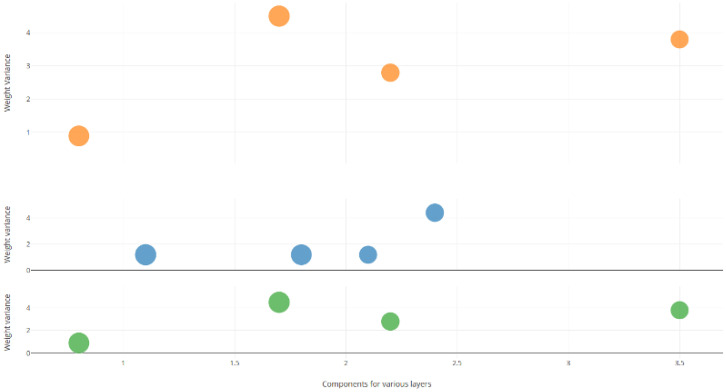
Visualization: Depiction of components based on weight variance for IRIS dataset with three classes.

A component is a group of weights that are extracted based on the model presented earlier. Weights are extracted as components from the two, three, and four layers of the five-layer neural network, and the component values based on variance are presented in Figure 6. Note that the experiment was conducted using a correlated feature dataset, which achieved a maximum accuracy of 99.8%.

Figure 6 shows the number of components on the x-axis and variance on the y-axis. Note that the x-axis is scaled to fit the variance value to depict the graph accurately.

The components in the middle layer denoted by blue dots show a near-ideal variance of ‘zero’, indicating that the components possess the majority of ‘knowledge’ in the form of feature components. The green dots show the components from the layer before the middle layer, and the orange dots denote the ones after. From Figure 6, it can be observed that the variance of the components extracted from weights keeps increasing, reaching an ideal (near zero) value at the middle layer, followed by a rise in the layer after.

To understand the changes precisely, it would be interesting to see the projection of weights.

**Figure 7:**
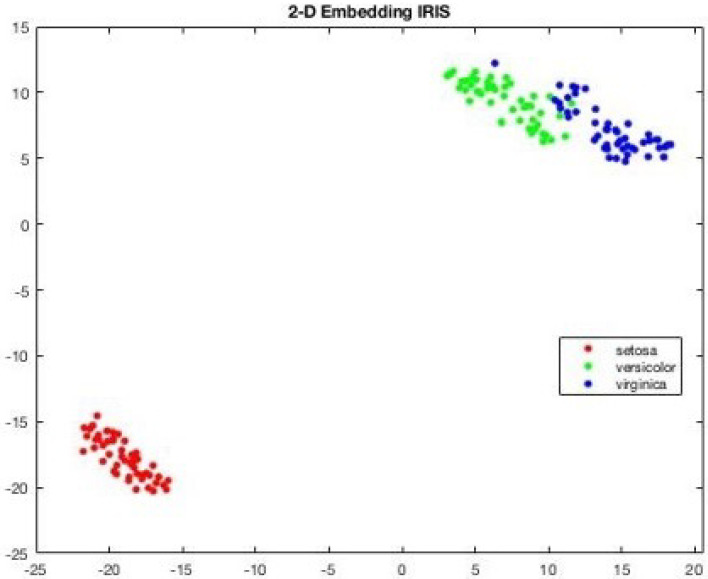
Visualization: 2D projection of IRIS dataset showing tone attribute far away, depicting the isolated setosa.

**Figure 8:**
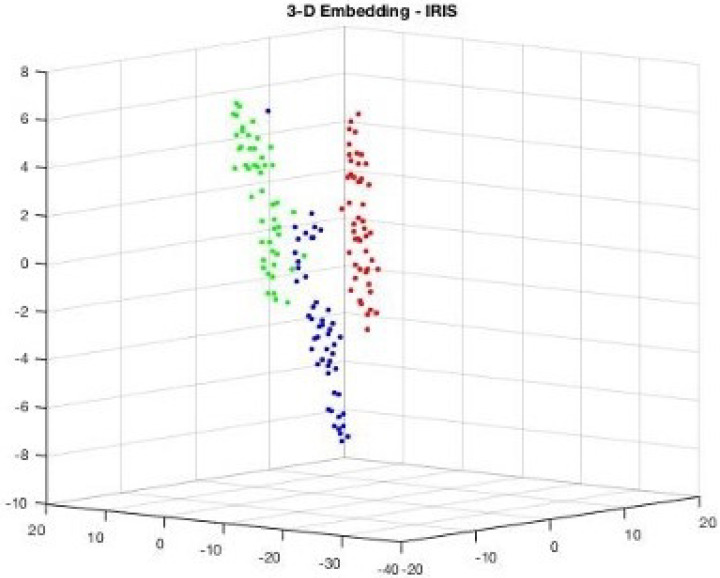
Visualization: 3D projection of IRIS dataset showing how attribute values are projected.

Consider Figure 7, the 2D projection of MIRIS1 classes from the dataset. Also, Figure 8 presents 3D projections of the same classes.

Figure 7 and Figure 8 show that there is a notable distance between the classes, and all the features are associated with each other based on the values that they belong to. Since the dataset MIRIS1 is modified to attain 100% accuracy, there are no isolated values.

**Figure 9:**
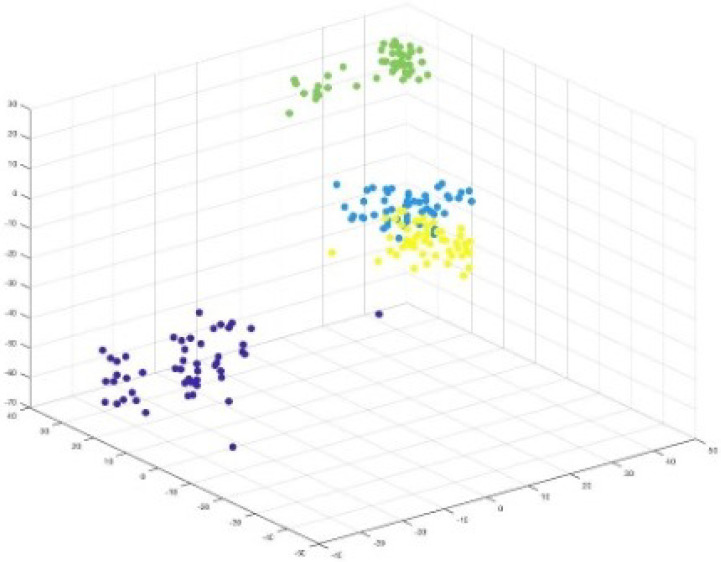
Visualization: Projection of MIRIS1 components representing overlapping features.

**Figure 10:**
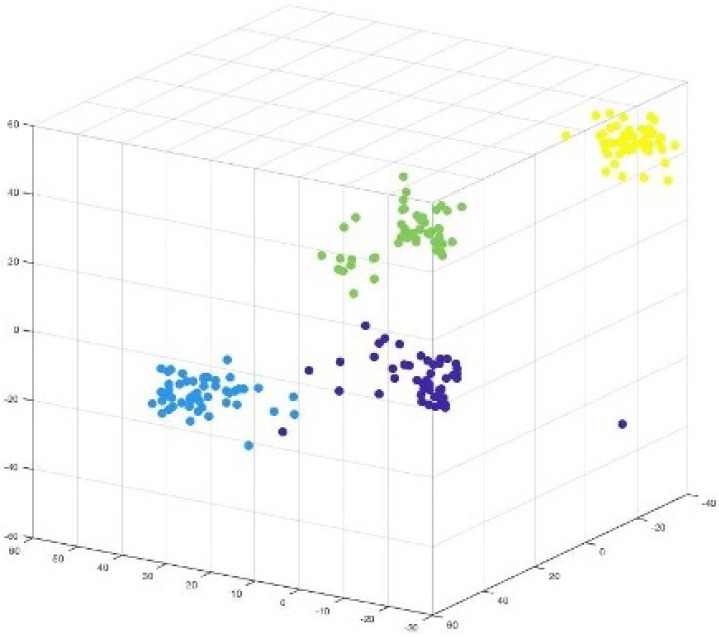
Visualization: Projection of MIRIS2 components with isolated feature (attribute).

The 2D and 3D projections of components (weights) from the middle layer are presented in Figure 9 and Figure 10, respectively.

The weight values extracted are colour-coded based on the components they belong to. Figure 9 clearly presents separated components, indicating how ‘similar’ features are grouped together. This is because each component consists of weights that belong to a feature, which are projections of input features.

It can be observed in Figure 7 and Figure 8 that two attributes (blue and green) are closer to each other, implying some similarity in their values. This is reflected in the visualization of components shown in Figure 9, which shows two component weights (yellow and blue) overlapping.

Figure 10 presents the 3D projection of components extracted from the MIRIS2 dataset with isolated attributes. The isolated attributes are reflected in the form of the isolated weight values of the components, which are presented in Figure 10 in yellow. Therefore, it could be concluded from the experiments that the input features are reflected in the neural network weights, which can be extracted in the form of components to show how features are represented in neural network weights.

### 4.3. Limitations

The proposed model is not exactly an FA- or PCA-based model but a combination of both based on factors like dataset type, number of components, etc. The experimental results are evaluated through statistical approaches (PCA and FA) and statistical parameters (*t*-test and RMSE). Although diversified datasets were used in the experiments, it is noteworthy to mention this as a limitation of this research that could provide a better method for future work. The evaluation of the component model is based on the fact that the features present in the input are relevant and have a direct association with the class label. In the case of noise data, component extraction could produce limited accuracy. Also, in the case of complex datasets, like high-dimensional datasets or more confusing datasets, it would be interesting to assess the efficiency of the proposed model. The model is also limited by overlapping features in the limited datasets. The model needs to be tested on high-dimensional images and other multimedia datasets. The FA and PCA models expect the dataset to possess statistically measurable variance. If a dataset that challenges PCA was used in the proposed model, it would be interesting to see how the component model would react.

## 5. Conclusions

This research proposes a novel component-based model to extract weights associated with knowledge or learning of features. The proposed model is based on the fact that the input features are represented in the form of a set of weights inside the layers of a neural network. Also, this work, by extracting the group of weights in the form of a component, enables the establishment and reiteration of the relationship between input features and neural network weights through experimental evaluation.

The IRIS dataset, widely accepted for experiments, is used in conjunction with four modified versions. Classification experiments are conducted using neural networks with four different topologies: 3, 5, 9, and 13 layers. t-SNE is used to visualize the input features and the extracted weight components. The results show that changes in the input features are clearly reflected in the components when visualized.

### Future Work

The proposed component model clearly depicted the features, reiterating the fact that input features are ‘learnt’ by neural networks through training, and they exist in the neural network weights, which is considered ‘knowledge’. From here, it will be interesting to see how the proposed model can be used to extract components from complex and high-dimensional datasets, as well as how these components can be used for transfer learning to improve efficiency. In addition, the extracted components are based on variance. However, determining the number of components is also important. It would be appropriate to develop a defined theory for determining the number of components and how they can be transferred to another neural network. Also, the model needs to be tested on complex, high-dimensional benchmark datasets to determine the effectiveness of the model.

## Figures and Tables

**Table 1:** Experimental results: Classification results with RMS error, accuracy, and *t*-test on IRIS dataset.

SNo	Layers	RMS Error	Accuracy	*t*-Test
1	3	2.64	73.8	0.023
2	5	1.9	79.0	0.019
3	9	0.41	84.5	0.013
4	13	5.53	27.1	0.24

**Table 2:** Experimental results: Classification results with RMS error, accuracy, and *t*-test on modified IRIS Dataset-1.

SNo	Layers	RMS Error	Accuracy	*t*-Test
1	3	0.024	98.6	0.02
2	5	0.0092	99.8	0.011
3	9	0.48	88.3	0.02
4	13	3.21	41.8	0.89

**Table 3:** Experiment Results: Classification results with RMS error, accuracy, and *t*-test on modified IRIS Dataset-2.

SNo	Layers	RMS Error	Accuracy	*t*-Test
1	3	5.5	58.4	0.12
2	5	4.24	43.7	0.18
3	9	4.09	45.5	0.06
4	13	11.9	16.4	0.08

**Table 4:** Experimental results: Classification results with RMS error, accuracy, and *t*-test on modified IRIS Dataset-3.

SNo	Layers	RMS Error	Accuracy	*t*-Test
1	3	7.43	28.9	0.069
2	5	4.44	43.8	0.072
3	9	4.682	45.3	0.012
4	13	13.81	11.2	0.63

## Data Availability

Data are contained within the article.
